# Myofascial Tissue and Depression

**DOI:** 10.1007/s10608-021-10282-w

**Published:** 2021-12-21

**Authors:** Johannes Michalak, Lanre Aranmolate, Antonia Bonn, Karen Grandin, Robert Schleip, Jaqueline Schmiedtke, Svenja Quassowsky, Tobias Teismann

**Affiliations:** 1 Department of Psychology and Psychotherapy, Witten/Herdecke University, Alfred-Herrhausen-Straße 50, 58448 Witten, Germany; 2 Department for Conservative and Rehabilitative Orthopedics, Technical University Munich, Georg-Brauchle-Ring 60/62, 80992 München, Germany; 3 Department for Medical Professions, DIPLOMA University of Applied Sciences, Am Hegeberg 2, 37242 Bad Sooden-Allendorf, Germany; 4 Department of Clinical Psychology and Psychotherapy, Ruhr-Universität Bochum, Massenbergstraße 9, 44787 Bochum, Germany

**Keywords:** Embodiment, Depression, Myofascial tissue, Memory bias

## Abstract

**Background:**

The myofascial system plays a fundamental role in the mechanics of the body, in body tension regulation and the etiology of pathological states like chronic pain. Moreover, it contains contractile elements and preliminary evidence suggests that its properties are linked to psychological factors. The aim of the present research was to investigate characteristics of the myofascial tissue in patients with Major Depressive Disorder (MDD) and to examine whether the state of the myofascial tissue causally affects pathopsychological processes in MDD.

**Methods:**

In Study 1, stiffness and elasticity of the myofascial tissue of 40 inpatients suffering from MDD measured with a tissue compliance meter were compared with those of 40 matched never-depressed participants. In Study 2, 69 MDD patients were randomly assigned to single-session self-myofascial release intervention (SMRI) or a placebo intervention. Effects on memory bias and affect were investigated.

**Results:**

Results showed that MDD patients displayed heightened stiffness and reduced elasticity of the myofascial tissue and that patients in the SMRI group showed a reduced negative memory bias and more positive affect compared to patients in the placebo condition.

**Conclusions:**

The preliminary results of our studies indicate that the myofascial tissue might be part of a dysfunctional body-mind dynamic that maintains MDD.

**Supplementary Information:**

The online version contains supplementary material available at 10.1007/s10608-021-10282-w.

## Introduction

Major depression disorder (MDD) is associated with significant suffering and impairment for the depressed individuals and their families. Moreover, MDD leads to substantial social costs (König et al., 2020). Given the high prevalence and its often recurring and chronic course it is important to develop comprehensive models of factors affecting the vulnerability and course of MDD. Up to now, psychological models of MDD have particularly focused on cognitive (e.g., rumination, negative cognitive style) and interpersonal factors (e.g., conflicts, diminished social support) (Hankin et al., 2018).

In addition to the well documented cognitive and interpersonal factors, some recent studies have examined the possible role of bodily processes as an etiology factor in MDD. These studies were inspired by accumulating evidence from basic research that motor displays affect emotional processes. This close interplay between motoric and emotional processes was, for example, documented in a recent meta-analysis including over 70 studies on experimental manipulations of motor displays in non-clinical participants (Elkjær et al., 2020). In these studies, participants were made to adopt for example an upright or slumped posture or to walk in a depressed or non-depressed style. The results of this meta-analysis have shown robust differences between contractive displays (e.g., slumped posture, sad gait pattern) and expansive displays (e.g., upright posture) for affective responses (e.g., power feeling and mood) and overt behavioral responses (e.g., risk taking) across different contexts, types of manipulation, and methods of measurement. Moreover, analyses of a subset of studies including a condition with neutral motor display indicate that the effects are driven by the absence of contractive motor displays rather than the presence of expansive displays.

Studies investigating the role of the motoric system in MDD have shown that depression is associated with a slumped posture, especially greater anterior head inclination and thoracic kyphosis (e.g., Canales et al., 2010; Wilkes et al., 2017) and gait pattern alternations (e.g., Michalak et al., 2009). Moreover, first experimental studies have documented that changing motor displays have causal effects on depression-related processes. Two studies have investigated the effects of short motor manipulations on memory bias in MDD. A biased memory is characterized by the tendency of depressed individuals to recall more negative than positive information in memory task. Non-depressed individuals show a bias in the positive direction, they tend to recall more positive than negative information. A negative memory bias is one of the most robust findings on cognitive processes in MDD (Gotlib & Joormann, 2010) and empirical studies have shown that a biased memory predicts the course of depression symptoms (Beeney & Arnett, 2008).

Michalak et al. (2014) showed that changing the sitting posture of inpatients suffering from MDD had effects on this biased recall of negative information. In this experiment participants sat either in a slumped (depressed) or in an upright (non-depressed) posture while imagining a visual scene of themselves in connection with the presentation of positive or depression-related words. After a distractor task an incidental recall test of these words was conducted. Patients sitting in an upright position showed an unbiased recall of positive and negative words while patients sitting in a slumped position showed the biased recall of negative words typical for depressed individuals.

Effects of motor manipulations on memory bias were also investigated in a study by Michalak et al. (2018). Patients suffering from MDD practiced either an upward-opening Qi Gong movement, which runs counter to the habitual slumped and downward depressive movement style, or a downward-closing Qi Gong movement. Again, an incidental recall of the cue words was conducted. Results showed that patients in the upward-opening movement condition in contrast to the downward-closing movement condition showed a more positively biased recall of affective words (i.e., they recalled more positive than negative words). Moreover, in this study effects of movements on overgeneral autobiographical memories were investigated. When depressed individuals are asked to remember events that refer to a particular time and place they often respond with overgeneral memories, referring to a whole class of events, or with references to semantically related content that does not include any autobiographical memory. An overgeneral memory is a stable cognitive characteristic of depressed individuals (see Williams et al., 2007 for a review). In the study of Michalak et al. (2018) patients in the upward-opening movement condition, in addition to a more positively biased memory, showed a reduced tendency to report overgeneral autobiographical memories.

The findings of these studies show that, comparable to the broad evidence base for the impact of manipulations of motor displays on emotional processes in non-clinical samples (Elkjær et al., 2020), motoric manipulations can also affect emotional processes in depressed individuals. Correspondingly, they support theoretical accounts that stress the relevance of the body in depression. Specifically, the Interacting Cognitive Subsystems approach (Teasdale & Barnard, 1993) proposes that proprioceptive and kinesthetic input from the body makes a direct and important contribution to emotional information processing in MDD. According to this theory, a depressive interlock configuration of bodily and cognitive feedback loops can become established that ‘locks’ subsystems into a self-perpetuating configuration that maintains depression. Self-perpetuating means that depressive cognitions lead to negative body displays (e.g., slumped posture, sad gait pattern) and in turn the negative body displays increase the tendency to think in a negative and depressive way, which leads to a vicious circle deepening the depressive states.

However, studies in basic as well as clinical research to date have only investigated the effects of short manipulations of motor displays. In our present research we investigated an important bodily system that plays a fundamental role in the mechanics of the body, in body tension regulation and the etiology of pathological states like chronic pain—the myofascial system. It builds a three-dimensional continuum of soft, loose and dense fibrous connective tissue containing collagen that permeates the body and enables all body systems to operate in an integrated manner. Classically it was proposed that the fascia tissue has merely a passive role in force transmission within the body. However, more recent research has shown that fascial tissue contains contractile elements enabling it to play a modulating role in force generation and also mechanosensory fine-tuning (Schleip & Klingler, 2019). Fascial stiffness and elasticity can be regulated within different time frames ranging from minutes to days and months. It is influenced by biochemical as well as biomechanical processes. The contractile activity of the fascial cells is biochemically influenced by the expression of various cytokines within the non-fibrous component of the extracellular matrix, also referred to as ground substance. For one of the cytokines involved in the transmission, TGF-β1, a clear influence of the autonomous nervous system on its activity has been documented (Bhowmick et al., 2009; Liao et al., 2014). Stress-related dysregulations of the autonomous nervous system (Alvares et al., 2016) and dysfunctions of the immune system, including elevated TGF-β1 levels (Davami et al., 2016; Lee & Kim, 2010), are features of MDD. Therefore, we expect that by these dysfunctions of the autonomous nervous and the immune system in individuals with MDD (e.g., elevated TGF-β1 levels with effects on contractile activity of the fascial cells)‚ characteristics of the myofascial tissue like heightened stiffness and reduced elasticity should result in individuals with MDD.

Moreover, in addition to this biochemical pathway we postulate a biomechanical pathway resulting in dysfunctional characteristics of the myofascial tissue in MDD. Individuals suffering from MDD often show greater anterior head inclination and thoracic kyphosis (Canales et al., 2010) and a slumped posture (Adolph et al, 2021; Michalak et al., 2009). In congruence with the well described *flexion-relaxation phenomenon* such postural changes tend to be associated with an enhanced mechanical loading of passive connective tissues on the posterior side of the trunk (Colloca & Hinrichs, 2005). Based on this mechanism, we expect that individuals suffering from MDD should show heightened stiffness and reduced elasticity of the myofascial tissue in the neck and upper back region. Moreover, since tightening the posterior neck and upper back has been described as a protective biological response to danger/stress (Bullock, 1984), the chronic stress associated with MDD might lead to a heightened stiffness and reduced elasticity especially in these regions of the body. To test the postulated dysfunctional characteristics of the myofascial tissue in MDD, in Study 1 we compared stiffness and elasticity of the myofascial tissue in the neck and upper back region of patients suffering from MDD with non-depressed control participants.

Moreover, we assumed that heightened stiffness and decreased elasticity of the myofascial tissue in the neck and upper back region might be part of depressive interlock configuration as the Interacting Cognitive Subsystem framework (Teasdale & Barnard, 1993) postulates. If heightened stiffness and decreased elasticity of the myofascial tissue becomes chronic it might be part of the proprioceptive input from the body that constantly makes negative and depressogenic cognitive and emotional process more accessible and therefore contributes to the dynamics of establishing self-perpetuating body-mind configurations that maintain depression. Therefore, in Study 2 we were interested in the possible causal contribution of the myofascial tissue to depressive processes and investigated the effects of a single-session self-myofascial release intervention on affect and negative memory bias in patients suffering from MDD. Negative affect and the biased recall of negative information are both key maintaining factors in cognitive models of MDD (e.g., Gotlib & Joormann, 2010; Rehm & Naus, 1990) and empirical studies have shown that negative emotionality (Wilson et al., 2014) and biased memory predict the course of depression symptoms (Beeney & Arnett, 2008; LeMoult et al., 2017; LeMoult et al., 2017; Rude et al., 2002).

## Study 1

### Methods

#### Participants

Participants were 40 psychiatric inpatients and 40 never-depressed control participants matched for age, sex and body-mass-index (BMI). The inpatients were recruited from two adult psychiatric units. Depressed patients were included in the study if they met the criteria of the Diagnostic and Statistical Manual of Mental Disorder (4th TR edition; DSM-IV-TR; American Psychiatric Association, 2000) for a primary diagnosis of current MDD and had a score of 14 or more on the Beck Depression Inventory. Exclusion criteria were as follows: psychotic disorders, bipolar disorders, current substance-related disorders, diseases of the musculoskeletal system and back pain. Diagnoses were derived by trained raters with the German version of the Structured Clinical Interview for DSM-IV (Wittchen et al., 1997).

The control participants were recruited by means of public notices. They were included in the control group if they had no current or lifetime MDD diagnosis. Exclusion criteria were derived with the SCID. Moreover, participants with diseases of the musculoskeletal system or back pain were excluded.

In both groups 25 participants were female and 15 male. All participants lived in Germany. The groups did not differ significantly in age (depressed patients: *M* = 35.15, *SD* = 12.43, never-depressed: *M* = 31.50, *SD* = 10.53) or BMI (depressed patients: *M* = 24.31, *SD* = 3.07, never-depressed: *M* = 23.35, *SD* = 3.01) (all *p*s > 0.15). Depressed patients had significantly higher BDI-II scores than never-depressed control participants (depressed patients: *M* = 30.40, *SD* = 7.73, never-depressed: *M* = 2.72, *SD* = 3.35; *t*[78] = 20.79, *p* < 0.001).

Twenty-five out of 40 depressed patients had comorbid diagnoses (most of them anxiety disorders). Thirty-nine depressed patients were receiving antidepressant medication, most of them Serotonin-Reuptake Inhibitors or Serotonin-Noradrenalin-Reuptake Inhibitors.

#### Beck Depression Inventory

The Beck Depression Inventory (BDI-II; Beck et al., 1996, German version by Hautzinger et al., 2006) is a widely used 21-item self-report measure covering affective, cognitive, motivational, behavioral and biological symptoms of depression with good psychometric properties.

#### Measurement of Myofascial Tissue

We measured the stiffness and elasticity of the myofascial tissue in the neck and upper back region of our participants with an electronic tissue compliance meter (ETCM). This tool was custom-built (Chemnitz University of Technology, Chemnitz, Germany) as a new and improved version of the semi-electronic tissue compliance meter described by Wilke et al. (2018) as a valid and reliable measurement tool to evaluate muscle stiffness. The latter study reported an intraclass correlation coefficient for test–retest reliability of 0.84 for the ETCM, indicating an excellent reliability.

In contrast to the semi-electronic tissue compliance meter, the ETCM incorporates an electronic registration of the measured force and strain values, permitting a resolution of 0.001 Newton and 0.01 mm as the smallest differences (compared with 0.1 Newton and 0.1 mm in the semi-electronic tissue compliance meter). Successful applications of the ECTM in scientific in vivo investigations in humans include a recent comparative assessment of the stiffness of the plantar fascia and heel pad (Holowka et al., 2019) and a recent evaluation of the stiffness of three different back muscles (Kett et al., 2020). For a more detailed technical description of the ETCM see Kett et al. (2020).

For the ETCM measurements in this study a fixed indentation depth of 8 mm was chosen. Three assessments were conducted at each location within a period of 30 s in which the center piece of the tool indented the tissue up to a depth of 8 mm and the maximum tissue resistance was measured in N at each indentation. The maximum resistance at the first indentation divided by the indentation depth (8 mm) was used as the stiffness value of this location, whereas the difference in resistance between the first and last indentation was used as an indicator of the tissue elasticity (reverse of viscolestic relaxation) (Rätsep et al., 2011). A difference of 0% would be interpreted as maximum elasticity (100%), whereas a difference of 100% between the first and last indentation would be interpreted as minimum elasticity. The location of the exact measurement point on the upper trapezius muscle was determineed according to Heizelmann et al. (2017). In addition, a location 2 cm below the inferior edge of the scapula on the thoracic back was used as an additional measurement site. All ETCM measurements were conducted at four locations: right trapezius, left trapezius, right thoracic back, left thoracic back. For the stiffness values as well as for elasticity, the mean of all four locations was used as a final value for each patient.

## Results

Descriptive statistics for elasticity and stiffness of the myofascial tissue in the depressed and never-depressed group can be found in Table 1. The correlation between stiffness and elasticity was r = 0.66 (p < 0.001) in the depressed group and r = 0.67 (p < 0.001) in the never-depressed group.

**Table 1:** Descriptive statistics (Study 1)

	Depressed patients (*n* = 40)	Never-depressed control participants (*n* = 40)
Stiffness: *M* (*SD)*	2.55 (1.02)	1.96 (0.82)
Elasticity: *M* (*SD)*	0.56 (0.42)	0.33 (0.25)

To test for group differences we conducted a multivariate analysis of variance (MANOVA) across the two groups (depressed vs. non-depressed) with elasticity and stiffness of the myofascial tissue as the two dependent variables. There was a significant multivariate effect of group (Wilks’s Lambda = 0.88), *F*(2, 77) = 5.13, *p* < 0.01, η^2^_p_ = 0.12, 90% CI (0.02—0.22) which was reflected in significant univariate group effects for elasticity (*F*[1,78] = 8.47, *p* < 0.01, η^2^_p_ = 0.10, 90% CI [0.02—0.21]) and stiffness (*F*[1,78] = 8.73, *p* < 0.01, η^2^_p_ = 0.10, 90% CI [0.02—0.21]). To test whether the small group differences in age and BMI that we descriptively observed might have affected our results, we conducted a multivariate analysis of covariance (MANCOVA) with age and BMI as covariates. In this MANCOVA the significant difference between the depressed and non-depressed group remained intact (for details see supplement).

The correlation between depression severity (BDI-II-scores) and stiffness was *r* = 0.32, *p* < 0.01, between depression severity and elasticity was *r* = 0.32, *p* < 0.01.

## Discussion

As expected patients with MDD and never-depressed control participants differed in the characteristics of the myofascial tissue. Depressed patients showed higher stiffness and reduced elasticity of the myofascial tissue. Since fascial tissue is involved in the modulation of force generation and also mechanosensory fine-tuning, long-term dysfunction in this tissue represented by stiffness and reduced elasticity might lead to chronically intensified body tension and reduced suppleness of the motoric system. This might be one of the reasons why depressed gait is characterized by reduced arm swing, a reduced vertical up-and-down dynamic (Michalak et al., 2009) and why depressed individuals show a slumped posture (Canales et al., 2010; Wilkes et al., 2017). Heightened body tension, depressed gait patterns and posture might then feedback into the psychological system and make negative cognitions and emotional states more accessible.

It should be noted that we measured characteristics of the myofascial tissue only in the upper back and neck region. It is unclear how dysfunctions in these regions are interconnected with the myofascial tissue of other body regions and how stiffness and reduced elasticity in the back and neck influence posture or more complex motor activity like gait in depressed individuals. Therefore, future studies should supplement the present findings by measuring characteristics of the myofascial tissue in other body regions and should also investigate effects of stiffness and reduced elasticity on posture and motoric activity of depressed individuals. Moreover, since the method used in our study focused on measuring stiffness and elasticity future research should use other methods like sonography and sonoelastography that permit a more fine-grained analysis of the characteristics of the fascia tissue (e.g., Langevin et al., 2009, Stecco et al., 2014). It is possible that such additional analyses may then be able to clarify more specifically to what degree the different tissue layers—including the dermis, subcutaneous connective tissue, Fascia profunda, and muscular layer—contribute to the increased stiffness and decreased elasticity observed in this study. Such contributions may involve—among other aspects—a change in the thickness, stiffness, regularity or in the shearing mobility of one or several tissue layers (Blain et al., 2019; De Coninck et al, 2018; Langevin et al., 2011).

Another issue that should be addressed in future research is the question whether the stiffness and reduced elasticity we observed is attributable to the slumped posture of depressed individuals (biomechanical explanation) or to a biochemical process in the myofascial tissues. This research should also utilize alternative methods to assess characteristics of the myofascial tissue (Schleip & Bartsch, 2021; Zügel et al., 2018). A future study using matched pair group of individuals with a slumped or forward head posture (e.g., people working in jobs with prolonged sitting) but without a diagnosis of MDD might also be useful for differentiating between a biomechanical and a biochemical causation of stiffness and reduced flexibility. Moreover, the role of medication and comorbid anxiety disorders should be addressed. Since many patients in our study were under medication and had comorbid anxiety disorders, future studies might include samples without medication and comorbid anxiety disorders.

To elucidate the possibility that the myofascial tissue is involved in a depressive interlock configuration that makes negative and depressogenic cognitive and emotional processes more accessible we conducted a second study involving an intervention targeting the myofascial tissue. Patients with MDD were randomized either to this intervention or to a placebo control condition and effects of the intervention on psychological factors involved in the maintenance of depression were investigated.

## Study 2

### Methods

#### Participants and Overview of Procedure

Sixty-nine psychiatric inpatients suffering from MDD were randomized either to a single-session self-myofascial release intervention (SMRI) (*n* = 38) or a placebo intervention (PI) (n = 31). We had aimed to collect data from 40 patients in each group. However, as a result of an early end to testing due to the Covid-19 pandemic the actual sample size was smaller. Post-hoc power analysis for a MANOVA with two groups and two response variables revealed that the actual samples size of the present study was large enough to detect an effect of at least medium effect size with Type I error rates set at 0.05 (two sided) and Type II error rates set at 0.80.

Patients were recruited from adult psychiatric units. Inclusion and exclusion criteria were the same as for the depressed sample in Study 1. Inclusion criteria: current major depressive episode as defined by the DSM-IV-TR and a score of 14 or more on the BDI-II (Beck et al., 1996; German version: Hautzinger et al., 2006). Exclusion criteria: psychotic disorders, bipolar disorders, current substance-related disorders, diseases of the musculoskeletal system and back pain. Inclusion and exclusion diagnoses were derived by trained raters with the German version of the Structured Clinical Interview for DSM-IV (Wittchen et al., 1997). Most patients (68%) had comorbid diagnoses (most of them anxiety disorders) and the majority (80%) were receiving treatment with antidepressant medication.

The SMRI group and the PI group did not differ in sex (SMR group: 19 female, 18 male, 1 non-binary; placebo group: 20 female, 11 male), age (SMR group: *M* = 37.89, *SD* = 13.74, placebo group: *M* = 35.55, *SD* = 11.17), BMI (SMR group: *M* = 26.32, *SD* = 5.81, placebo group: *M* = 24.74, *SD* = 2.82) or BDI scores (SMR group: *M* = 28.13, *SD* = 12.05, placebo group: *M* = 26.35, *SD* = 7.04) (all *p*s ≥ 0.15). All patients lived in Germany.

Participants completed the SMRI or PI in three phases: (1) They began the first phase by watching a short instruction video for the SMR or PI neck and back exercises. (2) In the second phase, while lying on a gymnastics mat, participants practiced the neck and back exercises for 30 s each using a foam roller while listening to the same audio instructions that had accompanied the video in the first phase. (3) After the training round, participants began the third phase in which the exercises were executed individually without audio instructions. Neck as well as back exercises were performed twice for 60 s each (i.e. 2 × 60 s SMRI or placebo neck exercise and 2 × 60 s SMRI or placebo back exercise). The order of neck and back exercises was randomized across participants. Between rounds, participants rested for 90 s (lying on the mat). During the resting phase, participants were directed to relax and were given the positive and negative words of the self-referential encoding task (see below). Finally, after the last exercise round the Positive and Negative Affect Schedule (PANAS) was administered. To prevent changes of posture from interfering with the after-effects of the exercises, participant continued to lie on the mat and PANAS instructions were given by video. The experimenter recorded participants’ verbal PANAS response.

#### Self-myofascial Release Intervention (SMRI)

Various methods to affect myofascial tissues have been developed, most of them are applied by physiotherapists (e.g., Ajimsha, 2011; Barnes, 1997). One method that can by applied independently of a therapist and individually by the patient is the use of a foam roller, which typically consists of a semi-rigid foam material. The foam roller makes it possible for the tissue to be rolled out using one’s own body weight. Through the rolling, the stiffness in myofascial tissues can be lowered (Kett et al., 2020) and in addition the sliding ability between adjacent fascial tissue layers can be increased (Griefahn et al., 2017; Krause et al., 2019). In addition, a dehydration and subsequent rehydration in the treated tissues during foam rolling has been proposed (Behm & Wilke, 2019). Moreover, Golgi receptors in the facia are stimulated imparting an inhibitory reflex that reduces muscle tone (Roylance et al., 2013). A randomized controlled trial has shown that a single SMRI session with a foam roller has short-term impact on the mobility of the fascia tissue. It improved the mobility of the thoracolumbar fascia (Griefahn et al., 2017).

**Fig. 1:**
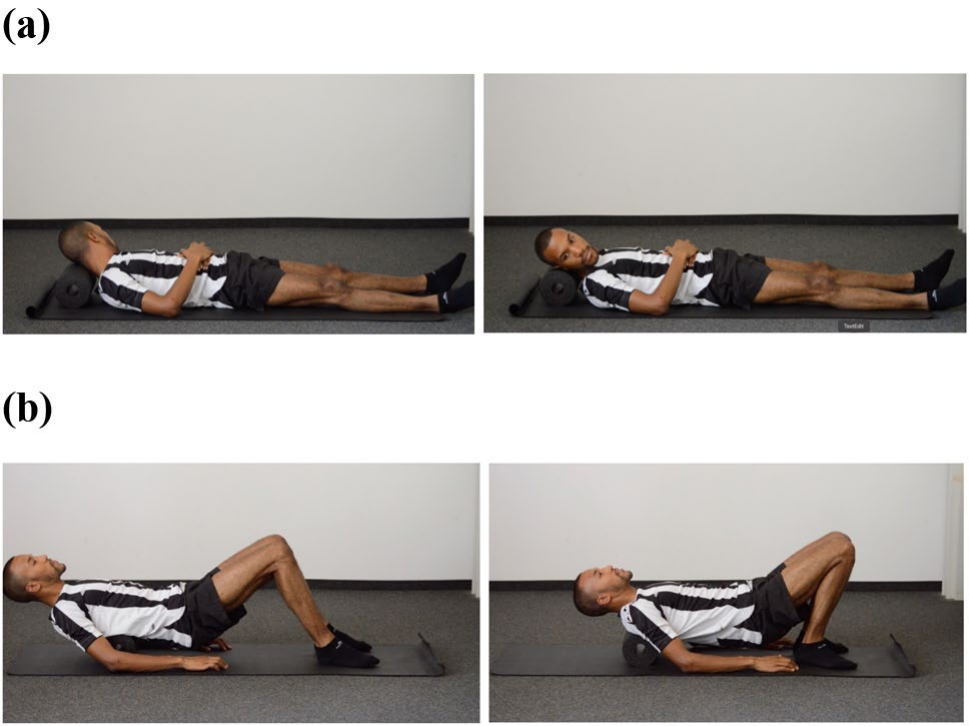
Self-myofascial release intervention for neck (**a**) and upper back (**b**)

Because Study 1 has shown that the myofascial tissue of the neck and upper back show reduced elasticity and higher stiffness in depressed patients we applied the SMRI with a foam roller to these body regions. The instruction videos were developed in accordance with the instruction manual for self-myofascial release intervention by Lukas (2012). During both SMRIs, participants were asked to lie on the mat. In the neck SMRI they were instructed to first rest their neck on the foam roller and then to roll their head slowly from right to left and back again continuously at an even pace for the duration of the exercise (see Fig. 1a). During the upper back SMRI, the participants were instructed to rest their back on the foam roller and slowly move the roller between their shoulders and the middle of their back evenly for the duration of the exercise (see Fig. 1b).

Because the exercises might induce pain, we instructed participants to allow discomfort, but to manage the level of pain so that it does not exceed 8 on a scale from 1 (slight tension, no pain) to 10 (extremely high tension, distressing pain). A rating of 8 was defined as very high tension that is bearable, slightly positive but tending to be uncomfortable; the participant can still talk to other people normally and has no physical, mental or emotional resistance to the tension.

#### Placebo Intervention

**Fig. 2:**
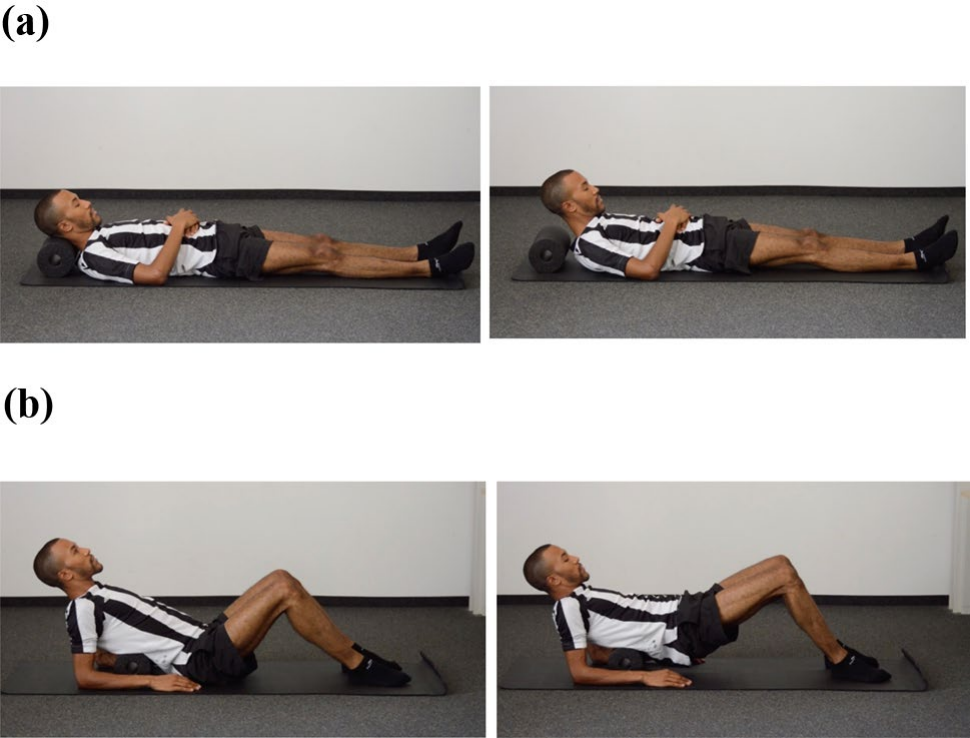
Placebo intervention for neck (**a**) and upper back (**b**)

Because SMRI is only effective through rolling out of the tissue but not just by pressure (MacDonald et al., 2013; Thömmes, 2014) we used a placebo intervention (PI) for both the neck and back without rolling movements. This placebo intervention was parallel to the SMRI in all aspects including the kind of instructions, exercise length, materials used, and the body position during the exercise. Participants in the PI used the same foam roller and mat during all exercises as participants of the SMRI. During both PIs, participants were asked to lie on the mat. In the neck PI they were instructed to first rest their neck on the foam roller and then to lift their head slowly and lower it back onto the roll continuously at an even pace for the duration of the exercise (see Fig. 2a). During the upper back PI, the participants were instructed to rest their back on the foam roller and slowly lift their torso from the mat and lower it again evenly for the duration of the exercise (see Fig. 2b). The instruction for managing discomfort and pain were identical to that given in the SMRI condition.

### Measures

#### Assessment of Memory Bias

To assess effects of the interventions on memory bias we adapted the self-referent encoding task (Ramel et al., 2007). In an initial encoding phase, participants, while resting in on the mat between the SMRI or PI exercise rounds, were presented a list of 10 positive and 10 negative words in random order by audio tape (negative words: bad, sorry, ugly, hurt, clumsy, helpless, angry, hopeless, guilty, lonely; positive words: pretty, secure, satisfied, proud, enthusiastic, happy, successful, relieved, interested, hopeful; words were taken from Williams & Broadbent, 1986). Directly after hearing each word, they were asked to respond out loud by saying either “yes” or “no” based on whether or not each word described them well. The participants were given 6 s for this response before the next word was presented. This phase was used to induce a self-referent encoding of the cue words.

Following all SMRI or PI exercises and after the participants completed the PANAS, an incidental recall of the cue words was conducted. Participants were asked (written instruction on the monitor) to recall out loud all words from the list of cue words they could remember and all answers were recorded by the experimenter. The number of positive and negative words correctly recalled was used as an indicator of biased memory in our analyses.

#### Positive and Negative Affect Schedule

To measure affect we used the Positive and Negative Affect Schedule (PANAS) state version (Watson et al., 1988; German version Krohne et al., 1996). It consists of two scales, one for positive and one for negative affect, each consisting of 10 adjectives with regard to which respondents rate their current mood on a Likert-type scale. It is a reliable (Cronbach’s α in the present study: PANAS positive scale: 86; PANAS negative scale: 81) and valid instrument to measure positive and negative affect.

#### Rating on Credibility of the Interventions

To assess the credibility of the SMRI and PI interventions we asked our participants after completion of the intervention: “At this point, how logical does the fascial intervention seem to you?”. The patients scored this item on a scale from 1 (not at all logical) to 9 (very logical).

#### Pain Rating

In addition to the scale that patients used to constantly monitor their pain level during the intervention (described above), we asked patients *after completion* of the intervention to rate retrospectively the level of pain provoked by the intervention on a scale ranging from 1 = “no pain at all” to 9 “very much pain”.

## Results

### Descriptive Statistics

Descriptive statistics as well as intercorrelations of affective variables can be found in Table 2. Most correlations are in the expected direction. Positive affective variables tend to correlate positively with other positive affective variables and negatively with negative affective variables. With only a few exceptions correlations tend to be in a low or medium range.

**Table 2:** Descriptive statistics and intercorrelations of variables (Study 2)

Variables	Self-myofascial release intervention (*n* = 38)						Placebo intervention (*n* = 30)					
	*M*	*SD*	1	2	3	4	*M*	*SD*	1	2	3	4
SRET positive words	3.63	3.16					2.35	2.32				
SRET negative words	1.55	1.45	− 0.08				2.87	2.42	− 0.07			
PANAS positive	2.76	0.58	0.38*	− 0.32*			2.37	0.64	0.68***	0.24		
PANAS negative	1.78	0.62	− 0.34*	0.30	− 0.50**		1.90	0.59	− 0.27	0.08	− 0.27	
BDI score	28.13	12.05	− 0.24	0.10	− 0.28	0.14	26.35	7.04	− 0.43*	0.01	− 0.40*	0.39*

SRET positive word = number of positive words recalled in the self-reference encoding task; SRET negative word = number of negative words recalled in the self-reference encoding task; PANAS positive = positive affect in the Positive and Negative Affect Schedule; PANAS negative = negative affect in the Positive and Negative Affect Schedule; BDI score: Beck Depression Inventory total score

We checked whether SMRI and PI differed in credibility or levels of pain induced by the intervention. Credibility ratings did not differ between conditions (SMRI condition *M* = 6.18, *SD* = 1.78; PI condition: *M* = 5.71, *SD* = 2.22; *t*[67] = − 0.98, *ns*). However, pain levels were higher during the SMRI than during PI (SMRI condition *M* = 4.29, *SD* = 2.17; PI condition: *M* = 2.90, *SD* = 1.66; *t*[67] = 2.98, *p* < 0.01, *g* = 0.79, 95% CI [0.22–1.20]).

### Effects of the SMRI on Biased Memory

To test whether SMRI and PI affected biased memory of the depressed patients differently, we conducted a MANOVA across the two groups (SMRI vs. PI) with the number of positive and the number of negative word recalled in the self-reference encoding tasks as the two dependent variables. There was a significant multivariate effect of group (Wilks’s Lambda = 0.86), *F*(2, 66) = 5.28, *p* < 0.01, η^2^_p_ = 0.14, 90% CI (0.02–0.25) which was reflected in a significant univariate group effect for negative words (*F*[1,67] = 7.86, *p* < 0.01, η^2^_p_ = 0.11, 90% CI [0.02–0.23]) and a statistical trend for positive words (*F*[1,67] = 3.52, *p* < 0.1, η^2^_p_ = 0.05, 90% CI [0.00–0.15]). Compared to depressed patients in the PI condition, those in the SMRI recalled a smaller number of negative words and showed a trend to recall more positive words. To test whether difference in pain induced by the intervention and credibility of interventions affected the results, we used a MANCOVA controlling for level of pain and for credibility. This MANCOVA revealed that controlling for pain and credibility did not change the pattern of results (for details see Supplement).

### Effects of the SMRI on Positive and Negative Affect

We also observed effects of the SMRI intervention on affect. A MANOVA across the two groups (SMRI vs. PI) with the PANAS positive affect and PANAS negative affect as the two dependent variables showed a significant multivariate effect of group (Wilks’s Lambda = 0.91), *F*(2, 66) = 3.47, *p* < 0.05, η^2^_p_ = 0.10, 90% CI (0.01–0.20) which was reflected in significant univariate group effect for PANAS positive mood (*F*[1,67] = 6.99, *p* < 0.01, η^2^_p_ = 0.09, 90% CI [0.01–0.21]). No significant univariate group differences were observed for PANAS negative affect (*F*[1,67] = 0.68, *ns*, η^2^_p_ = 0.01, 90% CI [0.00 – 0.08]). Depressed patients in the SMRI had a more positive mood than those in the PI condition. Controlling for level of pain and for credibility of the intervention in a MANCOVA did not change this pattern of results (for details see Supplement).

## Discussion

The results of Study 2 showed that an intervention targeting the myofascial tissue had effects on memory bias and affect of depressed individuals. Patients in the SMRI recalled a smaller number of negative words and had more positive affect compared to patients in the placebo condition. The mostly low or medium correlations between the outcome variables of Study 2 indicate that the different measures we used (i.e., memory bias, positive and negative affect,) tap different affective dimensions. Therefore SMRI seems to have effects on a relatively broad spectrum of affective dimensions.

The differences between the SMRI and PI that we observed cannot be attributed to the credibility of the intervention since credibility was comparable in both conditions and controlling for credibility in the MANCOVAs did not change the results. Moreover, controlling for pain levels did not change the results. This is a remarkable result because the SMRI induced more pain than the PI but produced more positive affective outcomes. That this effect is attributable to the operation of opponent processes (i.e., affective/hedonic contrasts induced by a negative stimulation, Solomon, 1980) is rather unlikely since our results survived when controlling for pain levels in the MANCOVAs. Rather, these results indicate that the effects of the SMRI on the myofascial tissue are responsible for our results. Since other research has shown that single-session SMRIs impacts the functional characteristics of the fascia tissue (Griefahn et al., 2017) it seems plausible that higher elasticity/reduced stiffness of the fascial tissue induced by the SMRI might be responsible for the effects on affect observed in our study. The effect might be explained by the somato-sensory input associated with the higher elasticity/reduced stiffness of the myofascial tissue induced by the SMRI. Stiffness and reduced elasticity might be biologically associated with states of heightened danger and stress. If stiffness decreases and elasticity increases by the SMRI, this might be a somato-sensory signal of reduced danger and stress. This in turn might lead to a more positive emotional state and a more positive memory processing leading to a de-escalation of the depressive mind–body interlock configuration postulated by the Interacting Cognitive Subsystems approach (Teasdale & Bernard, 1993).

However, one limitation of our study was that the effect of the SMRI on characteristics of the myofascial tissue (e.g., elasticity or stiffness) was not directly measured. We did not include this measure to reduce time burden and stress for our vulnerable participants. An augmenting influence of self-myofascial release on passive as well as active joint range of motion has been described (Skinner et al., 2020; Wilke et al., 2020). In addition, a short term reduction in myofascial stiffness has been documented as a response to a self-myofascial treatment of human back muscles (Kett et al., 2020). The latter study described a reduction in elastic modulus of the upper trapezius (in addition to other paraspinal muscles) in response to an 8-min foam roller self-massage in sedentary office workers. However, studies exploring foam rolling applications on the upper leg reported either no change (Pepper et al., 2021) or mixed results (Mayer et al., 2020) in terms of stiffness changes in response to their specific self-myofascial treatments. Our investigation followed the application protocol as well as the assessment technology as described by Kett et al. (2020) for the upper trapezius region to a high degree. It could be expected that the biomechanical tissue changes in this region reported in this study are very similar to our study. Nevertheless, further research is necessary to determine in which body regions and/or under what circumstances self-myofascial release treatments induce a change in the biomechanical tissue properties. Therefore, future research on the effects of SMRI should consider including the measures of the state of the myofascial tissue to directly link changes in myofascial tissue to changes in depressive processes.

Moreover, we cannot exclude that subtle difference between the SMRI and the placebo condition might be responsible for our results. We controlled for pain and credibility in our analyses. Furthermore, both SMRI and the control intervention included physical activity. However, it cannot completely be ruled out that subtle differences in level of physical activity might have influenced our results. Therefore futures studies should measure the physiological activity level produced by the SMRI and control condition. In addition, the role of the autonomous arousal stages on the effects we observed should be investigated. A strong mechanical stimulation of group II and III somatic afferent fibers has been shown to induce a short-term sympathetic activation, followed by a subsequent—and longer lasting—augmentation of vagal nerve activation (Terui & Koizumi, 1984). Future research with measures of autonomic responses should elucidate whether this mechanisms contributes to the effects of the SMRI on memory bias and affect.

Moreover, it should be noted that a single-session SMRI has only transient effects on fascial tissue. More enduring changes of the *structure* of the fascial tissue that lead to permanent heightened elasticity and reduced stiffness need more extensive training of up to 3 months (Bohm et al., 2015; Miller et al., 2005). Therefore, future research should investigate the effect of more extended SMRI on MMD.

A rather puzzling result was that the multivariate group effect observed in memory bias was particularly driven by group differences in the recall of *negative* words (and not positive words), while the multivariate group effect in affect was particularly driven by group differences in *positive* affect (and not negative affect). This difference is difficult to interpret and future research should investigate whether more extensive SMRI might lead to effects that span both positive and negative dimensions of memory bias and affect, respectively.

Taken as a whole the results of Study 2 showed that a self-administered intervention targeting the myofascial tissue can lead to changes in processes relevant in the etiology of MDD. The SMRI was relatively short and restricted to one session. Nevertheless, even this short intervention produced effects in memory bias and affect of moderate effects sizes.

## General Discussion

The aim of our present research was to investigate whether the myofascial tissue might contribute to the dynamics of establishing self-perpetuating mind–body interlock configurations that maintain depression. In Study 1 we observed that patients suffering from MDD, compared to non-depressed control participants, showed reduced elasticity and heightened stiffness of the myofascial tissue in the neck and upper back. Moreover, results of Study 2 indicate that changes in the myofascial tissue achieved by a SMRI can causally affect important pathopsychological processes involved in the maintenance of MDD. Therefore, the results of our present research indicate that characteristics of the myofascial tissue might be part of a depressive interlock configuration of bodily and psychological processes (Teasdale & Barnard, 1993) that ‘lock’ subsystems into a self-perpetuating configuration that maintains depression. Stiff and inflexible myofascial tissue seems to contribute to reduced positive affect and heightened accessibility of negative memories which in turn might increase stress that could further increase stiffness and reduce elasticity of the tissue. While the latter part of these body–mind vicious circles (effects of stress on characteristics of the tissue) was not tested in the present research our results support the notion that the myofascial tissue can affect emotional processes. Thus, our results are consistent with a large number of studies showing that bodily processes like movement patterns and posture affect emotional processes in non-clinical samples (Elkjær et al., 2020) and emerging evidence that bodily processes might also be relevant in the etiology of MDD (e.g., Michalak et al., 2014).

Moreover, the results of the present research correspond with phenomenological approaches to depression (Fuchs, 2013; Fuchs & Schlimme, 2009, Ratcliff, 2015). Phenomenological approaches stress that individuals suffering from depression often report a sense of bodily rigidity. This rigidity is manifested in bodily feelings like having a tire around the chest, a sense of pressure in the head or a general sense of tightness of the body. Instead of expressing the self, in depression the body is thus turned into a barrier to impulses directed to the environment. Through its rigidity, the body no longer gives access to the world, but stands in the way as an obstacle, separated from its surroundings. Phenomenological approaches posit that such an altered feeling of “being-in-the-world” (separated instead of connected to the world) is a core aspect of the phenomenon of depression. It can be speculated that the heightened stiffness and reduced elasticity of the myofascial tissue we observed in our research is a physiological correlate for the sense of rigidity that is a core characteristic of the depressed feeling of “being-in-the-world”. However, it should be noted that although some authors postulate an important role of the myofascial tissue in proprioception and body awareness (e.g., Langevin, 2021), no study yet has directly examined whether people can consciously detect the level of and degree of change in tissue stiffness and elasticity.

Future research should extend the findings of the present research by investigating a more fine-grained analysis of the role of the myofascial tissue in MDD and other psychological disorders. For example, it should be investigated whether stiffness and reduced elasticity can be found only in the neck and upper body region or whether the tissue in other regions of the body also shows these characteristics. Moreover, future research should investigate whether dysfunctions of the myofascial tissue are specific to MDD or whether they can also be observed in other psychological disorders. In addition, the predictive utility of characteristics of the myofascial tissue in longitudinal studies on the course of depression should be examined. Furthermore, investigating the association between characteristics of the myofascial tissue and the subjective experience of the body reported in phenomenological approaches and the analysis of the interplay between characteristics of the myofascial tissue and deviations in the motoric system of patients with MDD (e.g., gait or posture, Michalak et al., 2009) might be valuable lines of research. Moreover, future research should elucidate the causes of the stiffness and reduced elasticity of the myofascial tissue in MDD. Besides research on biological factors that impact structure and function of the tissue (e.g., generic factors, hormones) it could be promising to examine the role of past experiences like critical life-events or childhood adversities in the formation of a body memory including stiff and inflexible myofascial tissue (Koch et al., 2012). A limitation of our studies is that data on racial/ethnic identification and socioeconomic status of the participants was not collected.

In addition to the contribution of our results to a deepened theoretical understanding of the etiology of MDD, they might also shed light on new perspectives on the treatment of this debilitating condition. Future research should investigate whether intervention affecting the myofascial tissue might help to deescalate possible dysfunctional body–mind vicious circles in MDD. They should investigate the effects of a series of SMRI. It would be useful to include objective outcome measures and the assessment of retention effects by follow-up measures in these studies. In addition to SMRI, a relatively broad spectrum of other interventions exists targeting the myofascial tissue. Fascial interventions applied by physiotherapists (e.g., Ajimsha, 2011; Barnes, 1997) are widely used. However, their possible role as a component in the treatment of MDD has not yet been studied. Moreover, Asian approaches like Yoga or some Qi Gong systems aim to improve elasticity of the fascial tissue by stretching or specific movement practices. There is preliminary evidence that Yoga as well as Qi Gong have effects in the treatment of MDD (e.g., Cramer et al., 2013; Liu et al., 2015). An advantage of SMRI, Yoga and Qi Gong is that they can be used regularly on a self-administered basis. It could be worthwhile to investigate the combination of these bodily approaches with classical treatment approaches for MDD (i.e., cognitive behavioral) in methodologically rigorous, randomized controlled trials.

In summary, the present study offers evidence that myofascial tissue might be involved in the etiology of depression and that it might be part of a dysfunctional body-mind dynamic that maintains MDD. However, it should be noted that we have broken new ground with our study and the results should therefore be considered preliminary. Replication studies using more sophisticated methodology are needed before firm conclusions can be drawn. Especially, a replication of Study 1 testing whether the heightened stiffness and reduced elasticity are specific to the neck and shoulder region or can also be also found in other regions and a replication of Study 2 that includes a direct measurement of stiffness/elasticity are needed. While keeping these limitations in mind, our hope is that our results might have the potential to deepening our theoretical understanding of MDD and might also inspire innovative approaches to the treatment of MDD which take into account the probably important role of bodily processes in the formation of this debilitating condition.

## Supplementary Information

Below is the link to the electronic supplementary material.

Supplementary file1 (DOCX 14 KB)

## References

[CR1] Adolph D, Tschacher W, Niemeyer H, Michalak J (2021). Gait patterns and mood in everyday life: A comparison between depressed patients and non-depressed controls. Cognitive Therapy and Research.

[CR2] Ajimsha MS (2011). Effectiveness of direct vs indirect technique myofascial release in the management of tension-type headache. Journal of Bodywork and Movement Therapies.

[CR3] Alvares GA, Quintana DS, Hickie IB, Guastella AJ (2016). Autonomic nervous system dysfunction in psychiatric disorders and the impact of psychotropic medications: A systematic review and meta-analysis. Journal of Psychiatry and Neuroscience.

[CR4] American Psychiatric Association (2000). DSM-IV-TR: Diagnostic and statistical manual of mental disorders.

[CR5] Barnes MF (1997). The basic science of myofascial release: Morphologic change in connective tissue. Journal of Bodywork and Movement Therapies.

[CR6] Beck AT, Steer RA, Brown GK (1996). Manual for the Beck Depression Inventory-II.

[CR7] Beeney J, Arnett PA (2008). Stress and memory bias interact to predict depression in multiple sclerosis. Neuropsychology.

[CR8] Behm DG, Wilke J (2019). Do self-myofascial release devices release myofascia? Rolling mechanisms: A narrative review. Sports Medicine.

[CR9] Bhowmick S, Singh A, Flavell RA, Clark RB, O'Rourke J, Cone RE (2009). The sympathetic nervous system modulates CD4(+)FoxP3(+) regulatory T cells via a TGF-beta-dependent mechanism. Journal of Leukocyte Biolology.

[CR10] Blain M, Bedretdinova D, Bellin MF, Rocher L, Gagey O, Soubeyrand M, Creze M (2019). Influence of thoracolumbar fascia stretching on lumbar back muscle stiffness: A supersonic shear wave elastography approach. Clinical Anatomy.

[CR11] Bohm S, Mersmann F, Arampatzis A (2015). Human tendon adaptation in response to mechanical loading: A systematic review and meta-analysis of exercise intervention studies on healthy adults. Sports Medicine Open.

[CR12] Bullock TH, Eaton R (1984). Comparative neuropathology of startle, rapid escape, and giant fiber-mediated responses. Neural mechanisms of startle behavior.

[CR13] Canales JZ, Cordás TA, Fiquer JT, Cavalcante AF, Moreno RA (2010). Posture and body image in individuals with major depressive disorder: A controlled study. Brazilian Journal of Psychiatry.

[CR14] Colloca CJ, Hinrichs RH (2005). The biomechanical and clinical significance of the lumbar erector spinae flexion-relaxation phenomenon: A review of literature. Journal of Manipulative and Physiological Therapeutics.

[CR15] Cramer H, Lauche R, Langhorst J, Dobos G (2013). Yoga for depression: A systematic review and meta-analysis. Depression and Anxiety.

[CR16] Davami MH, Baharlou R, Vasmehjani AA, Ghanizadeh A, Keshtkar M, Dezhkam I, Atashzar MR (2016). Elevated IL-17 and TGF-β serum levels: A positive correlation between T-helper 17 cell-related pro-inflammatory responses with major depressive disorder. Basic and Clinical Neurosience.

[CR17] De Coninck K, Hambly K, Dickinson JW, Passfield L (2018). Measuring the morphological characteristics of thoracolumbar fascia in ultrasound images: An inter-rater reliability study. BMC Musculoskeletal Disorders.

[CR18] Elkjær E, Mikkelsen MB, Michalak J, Mennin DS, O’Toole MS (2020). Expansive and contractive postures and movement: A systematic review and meta-analysis of the effect of motor displays on affective and behavioral responses. Perspectives on Psychological Science.

[CR19] Fuchs T (2013). Depression, intercorporeality and interaffectivity. Journal of Consciousness&nbsp;Studies.

[CR20] Fuchs T, Schlimme JE (2009). Embodiment and Psychopathology: A phenomenological perspective. Current Opinion in Psychiatry.

[CR21] Gotlib IH, Joormann J (2010). Cognition and depression: Current status and future directions. Annual Review of Clinical Psychology.

[CR22] Griefahn A, Oehlmann J, Zalpour C, von Piekartz H (2017). Do exercises with the foam roller have a short-term impact on the thoracolumbar fascia? A randomized controlled trial. Journal of Bodywork and Movement Therapies.

[CR23] Hankin BL, Young JF, Gallop R, Garber J (2018). Cognitive and interpersonal vulnerabilities to adolescent depression: Classification of risk profiles for a personalized prevention approach. Journal of Abnormal Child Psychology.

[CR24] Hautzinger M, Keller F, Kühner C (2006). Beck Depressions-Inventar (BDI-II). Revision.

[CR25] Heizelmann A, Tasdemir S, Schmidberger J, Gräter T, Kratzer W, Grüner B (2017). Measurements of the trapezius and erector spinae muscles using virtual touch imaging quantification ultrasound-elastography: A cross section study. BMC Musculoskeletal Disorder.

[CR26] Holowka NB, Wynands B, Drechsel TJ, Yegian AK, Tobolsky VA, Okutoyi P, Milani TL (2019). Foot callus thickness does not trade off protection for tactile sensitivity during walking. Nature.

[CR27] Kett AR, Sichting F (2020). Sedentary behaviour at work increases muscle stiffness of the back: Why roller massage has potential as an active break intervention. Applied Ergonomics.

[CR28] Koch SC, Fuchs T, Summa M, Müller C (2012). Body memory, metaphor and movement.

[CR29] König H, König HH, Konnopka A (2020). The excess costs of depression: A systematic review and meta-analysis. Epidemiology and Psychiatric Sciences.

[CR30] Krause F, Wilke J, Niederer D, Vogt L, Banzer W (2019). Acute effects of foam rolling on passive stiffness, stretch sensation and fascial sliding: A randomized controlled trial. Human Movement Science.

[CR31] Krohne HW, Egloff B, Kohlmann CW, Tausch A (1996). Untersuchungen mit einer deutschen Version der "Positive and Negative Affect Schedule"(PANAS). Diagnostica.

[CR32] Langevin HM (2021). Fascia mobility, proprioception, and myofascial pain. Reduced thoracolumbar fascia shear strain in human chronic low back pain. Life.

[CR33] Langevin HM, Fox JR, Koptiuch C, Badger GJ, Greenan-Naumann AC, Bouffard NA, Henry SM (2011). Reduced thoracolumbar fascia shear strain in human chronic low back pain. BMC Musculoskeletal Disorders.

[CR34] Langevin HM, Stevens-Tuttle D, Fox JR, Badger GJ, Bouffard NA, Krag MH, Henry SM (2009). Ultrasound evidence of altered lumbar connective tissue structure in human subjects with chronic low back pain. BMC Musculoskeletal Disorders.

[CR35] Lee H-Y, Kim Y-K (2010). Transforming growth factor-beta1 and major depressive disorder with and without attempted suicide: Preliminary study. Psychiatry Research.

[CR36] LeMoult J, Kircanski K, Prasad G, Gotlib IH (2017). Negative self-referential processing predicts the recurrence of major depressive episodes. Clinical Psychological Science.

[CR37] Liao MH, Liu SS, Peng IC, Tsai FJ, Huang HH (2014). The stimulatory effects of alpha1-adrenergic receptors on TGF-beta1, IGF-1 and hyaluronan production in human skin fibroblasts. Cell and Tissue Research.

[CR38] Liu X, Clark J, Siskind D, Williams GM, Byrne G, Yang JL, Doi SA (2015). A systematic review and meta-analysis of the effects of Qigong and Tai Chi for depressive symptoms. Complementary Therapies in Medicine.

[CR39] Lukas C (2012). Faszienbehandlung mit der Blackroll [Treatment of fascia with the blackroll].

[CR40] MacDonald GZ, Penney MD, Mullay ME, Cuconato AL, Drake CD, Behm DG, Button DC (2013). An acute bout of self-myofascial release increases range of motion without a subsequent decrease in muscle activation or force. Journal of Strength and Conditioning Research.

[CR41] Matt GE, Vázquez C, Campbell WK (1992). Mood-congruent recall of affectively toned stimuli: A meta-analytic review. Clinical Psychology Review.

[CR42] Mayer I, Hoppe MW, Freiwald J, Heiss R, Engelhardt M, Grim C, Hotfiel T (2020). Different effects of foam rolling on passive tissue stiffness in experienced and nonexperienced athletes. Journal of Sport Rehabilitation.

[CR43] Michalak J, Chatinyan A, Chourib H, Teismann T (2018). The impact of upward versus downward movement patterns on memory characteristics of depressed individuals. Psychopathology.

[CR44] Michalak J, Mischnat J, Teismann T (2014). Sitting posture makes a difference: Embodiment effects on depressive memory bias. Clinical Psychology & Psychotherapy.

[CR45] Michalak J, Troje NF, Fischer J, Vollmar P, Heidenreich T, Schulte D (2009). Embodiment of sadness and depression: Gait patterns associated with dysphoric mood. Psychosomatic Medicine.

[CR46] Miller BF, Olesen JL, Hansen M, Døssing S, Crameri RM, Welling RJ, Smith K (2005). Coordinated collagen and muscle protein synthesis in human patella tendon and quadriceps muscle after exercise. The Journal of Physiology.

[CR47] Quirin M, Bode RC (2014). An alternative to self-reports of trait and state affect. European Journal of Psychological Assessment.

[CR48] Ratcliffe M (2015). Experiences in depression: A study in phenomenology.

[CR49] Ramel W, Goldin PR, Eyler LT, Brown GG, Gotlib IH, McQuaid JR (2007). Amygdala reactivity and mood-congruent memory in individuals at risk for depressive relapse. Biological Psychiatry.

[CR50] Rätsep T, Asser T (2011). Changes in viscoelastic properties of skeletal muscles induced by subthalamic stimulation in patients with Parkinson's disease. Clinical Biomechanics.

[CR51] Rehm LP, Naus MJ, Ingram RE (1990). A memory model of emotion. Contemporary psychological approaches to depression.

[CR52] Roylance DS, George JD, Hammer AM, Rencher N, Fellingham GW, Hager RL, Myrer WJ (2013). Evaluating acute changes in joint range-of-motion using self-myofascial release, postural alignment exercises, and static stretches. International Journal of Exercise Science.

[CR53] Rude SS, Wenzlaff RM, Gibbs B, Vane J, Whitney T (2002). Negative processing biases predict subsequent depressive symptoms. Cognition & Emotion.

[CR54] Schleip R, Klingler W (2019). Active contractile properties of fascia. Clinical Anatomy.

[CR55] Schleip R, Bartsch K, Schleip R, Wilke J (2021). Mechanical assessment. Fascia in sport and movement.

[CR56] Skinner B, Moss R, Hammond L (2020). A systematic review and meta-analysis of the effects of foam rolling on range of motion, recovery and markers of athletic performance. Journal of Bodywork and Movement Therapies.

[CR57] Stecco A, Meneghini A, Stern R, Stecco C, Imamura M (2014). Ultrasonography in myofascial neck pain: Randomized clinical trial for diagnosis and follow-up. Surgical and Radiologic Anatomy.

[CR58] Teasdale JD, Barnard PJ (1993). Affect, cognition and change: Remodelling depressive thought.

[CR59] Terui N, Koizumi K (1984). Responses of cardiac vagus and sympathetic nerves to excitation of somatic and visceral nerves. Journal of the Autonomic Nervous System.

[CR60] Thömmes, F. (2014). *Faszientraining: Physiologische Grundlagen, Trainingsprinzipien, Anwendungen im Team- und Ausdauersport sowie Einsatz in Prävention und Rehabilitation [Fascia training: Physiological basics, training principles, applications in team and endurance sports as well as use in prevention and rehabilitation]*. Stiebner Verlag.

[CR61] Watson D, Clark LA, Tellegen A (1988). Development and validation of brief measures of positive and negative affect: The PANAS scales. Journal of Personality and Social Psychology.

[CR62] Wilke J, Müller AL, Giesche F, Power G, Ahmedi H, Behm DG (2020). Acute effects of foam rolling on range of motion in healthy adults: A systematic review with multilevel meta-analysis. Sports Medicine.

[CR63] Wilke J, Vogt L, Pfarr T, Banzer W (2018). Reliability and validity of a semi-electronic tissue compliance meter to assess muscle stiffness. Journal of Back and Musculosketal Rehabilitation.

[CR64] Wilkes C, Kydd R, Sagar M, Broadbent E (2017). Upright posture improves affect and fatigue in people with depressive symptoms. Journal of Behavior Therapy and Experimental Psychiatry.

[CR65] Williams JMG, Barnhofer T, Crane C, Hermans D, Raes F, Watkins ER, Dagleish T (2007). Autobiographical memory specificity and emotional disorders. Psychological Bulletin.

[CR66] Williams JMG, Broadbent K (1986). Distraction by emotional stimuli: Use of a stroop task with suicide attempters. British Journal of Clinical Psychology.

[CR67] Wilson S, Vaidyanathan U, Miller MB, McGue M, Iacono WG (2014). Premorbid risk factors for major depressive disorder: Are they associated with early onset and recurrent course?. Development and Psychopathology.

[CR68] Wittchen HU, Wunderlich U, Gruschwitz S, Zaudig M (1997). Strukturiertes Klinisches Interview für DSM-IV (SKID).

[CR69] Zügel M, Maganaris CN, Wilke J, Jurkat-Rott K, Klingler W, Wearing SC, Findley T, Barbe MF, Steinacker JM, Vleeming A, Bloch W (2018). Fascial tissue research in sports medicine: From molecules to tissue adaptation, injury and diagnostics: Consensus statement. British Journal of Sports Medicine.

