# Supplementary material for: Myofascial Tissue and Depression
Source: Cognit Ther Res. 2021 Dec 21;46(3):560–72. doi: 10.1007/s10608-021-10282-w (PMC8688142; doi:10.1007/s10608-021-10282-w)
Supplement: Supplementary file 1 — Supplementary file1 (DOCX 14 KB) [file 10608_2021_10282_MOESM1_ESM.docx]

**Supplemental**

**Study 1:**

**Multivariate analysis of covariance (MANCOVA) of group difference (depressed vs. non depressed) in flexibility and stiffness of the myofascial tissue controlling for BMI and age**

We tested group differences across groups (depressed vs. non depressed) in a MANCOVA with flexibility and stiffness of the myofascial tissue as the two dependent variables and with age and BMI as covariates. There was a significant multivariate effect of group (F[2,75] = 3.61, p < .05, η^2^_p_ = .09) as well as the significant univariate group effects for flexibility (*F*[1,76] = 6.42, *p* <.05, η^2^_p_ = .08) and stiffness (*F*[1,76] = 5.56, *p* <.05, η^2^_p_ = .07).

**Study 2:**

**MANCOVA of differences between SMRI and PI in biased memory controlling for credibility and pain levels**

We controlled for credibility and pain level (covariates) in this MANCOVA with number of positive words and number of negative words recalled as the dependent variables. There was a significant multivariate effect of group (SMRI vs. PI; Wilks’s Lambda = .84), *F*(2, 64) = 6.08, *p* < .01, η^2^_p_ = .16, which was reflected in significant univariate group effects for negative words (*F*[1,65] = 9.76, *p* <.01, η^2^_p_ = .13) and statistical trend for positive words (*F*[1,65] = 3.22, *p* <.1, η^2^_p_ = .05).

**MANCOVA of differences between SMRI and PI in explicit affect controlling for credibility and pain levels**

We controlled for credibility and pain level (covariates) in this MANCOVA with PANAS positive and PANAS negative affect as dependent variables. There was a significant multivariate effect of group (SMRI vs. PI; Wilks’s Lambda = .89), *F*(2, 64) = 3.86, *p* < .05, η^2^_p_ = .10, which was reflected in significant univariate group effect for PANAS positive affect (*F*[1,65] = 7.79, *p* <.01, η^2^_p_ = .11). No univariate significant group differences were observed for PANAS negative affect (*F*[1,65] = 1.65, *ns*, η^2^_p_ = .03).

**MANCOVA of differences between SMRI and PI in implicit affect controlling for credibility and pain levels**

We controlled for credibility and pain level (covariates) in this MANCOVA with IPANAT positive and IPANAT negative affect as dependent variables. There was no significant multivariate effect of group in the MANCOVA with IPANAT positive and IPANAT negative affect as dependent variables (SMRI vs. PI; Wilks’s Lambda = .94), *F*(2, 64) = 2.07, *ns*, η^2^_p_ = .06).
